# MCM10, a potential diagnostic, immunological, and prognostic biomarker in pan-cancer

**DOI:** 10.1038/s41598-023-44946-2

**Published:** 2023-10-17

**Authors:** Dengwang Chen, Na Zhong, Zhanwen Guo, Qinglu Ji, Zixuan Dong, Jishan Zheng, Yunyan Ma, Jidong Zhang, Yuqi He, Tao Song

**Affiliations:** 1 Department of Immunology, Zunyi Medical University, Zunyi, China; 2 Collaborative Innovation Center of Tissue Damage Repair and Regeneration Medicine, Zunyi Medical University, Zunyi, China; 3 Special Key Laboratory of Gene Detection and Therapy of Guizhou Province, Zunyi Medical University, Zunyi, China; 4 School of Pharmacy, Zunyi Medical University, Zunyi, China; 5 School of Medical Information Engineering, Zunyi Medical University, Zunyi, China

**Keywords:** Cancer, Computational biology and bioinformatics, Immunology, Oncology

## Abstract

Microchromosome maintenance (MCM) proteins are a number of nuclear proteins with significant roles in the development of cancer by influencing the process of cellular DNA replication. Of the MCM protein family, MCM10 is a crucial member that maintains the stability and extension of DNA replication forks during DNA replication and is significantly overexpressed in a variety of cancer tissues, regulating the biological behaviour of cancer cells. But little is understood about MCM10’s functional role and regulatory mechanisms in a range of malignancies. We investigate the impact of MCM10 in human cancers by analyzing data from databases like the Gene Expression Profiling Interaction Analysis (GEPIA2), Genotype-Tissue Expression (GTEx) and The Cancer Genome Atlas (TCGA), among others. Possible relationships between MCM10 and clinical staging, diagnosis, prognosis, Mutation burden (TMB), microsatellite instability (MSI), immunological checkpoints, DNA methylation, and tumor stemness were identified. The findings demonstrated that MCM10 expression was elevated in the majority of cancer types and was connected to tumor dryness, immunocytic infiltration, immunological checkpoints, TMB and MSI. Functional enrichment analysis in multiple tumors also identified possible pathways of MCM10 involvement in tumorigenesis. We also discovered promising MCM10-targeting chemotherapeutic drugs. In conclusion, MCM10 may be a desirable pan-cancer biomarker and offer fresh perspectives on cancer therapy.

## Introduction

One of the top causes of death globally and a significant danger to human health is cancer^1^. Worldwide, cancer incidence and fatality rates are rising quickly^2,3^. The exceedingly difficult tumorigenesis process and dismal prognosis continue to be major obstacles in the fight against cancer. Pan-cancer analysis can help us comprehend the similarities between various cancer types and offer fresh perspectives for treating pan-cancer^4^. Exploring novel candidate genes for the early diagnosis and prognostic prediction of different cancers is therefore urgently needed. Pan-cancer analysis is crucial and feasible for the assessment of newly discovered cancer-associated genes^5^.

DNA replication stress is the stalling or slowing down of the replication process as a result of several processes (such as DNA strand breakage, a deficiency of nucleotides, etc.) that obstruct the regular replication process. Recently, DNA strand break repair mechanisms have drawn a lot of interest as possible therapeutic targets^6^. MCM10 was originally identified as a factor required for the replication of the genome and the stable maintenance of small chromosomes^7,8^, and is essential for replication-derived triggering^9^. However, recent studies have found that MCM10 is relevant to the growth of several tumors including breast^10^, liver^11^ and ovarian cancers^12^. Notably, MCM10 is highly expressed in almost all cancer types. Most importantly, MCM10 depletion reduced the growth of cancer cells but not normal cells. Moreover, our ongoing studies also suggest that MCM10 may act as an oncogene that is critical for the malignant proliferation of tumor cells^13^. Despite these results, more investigation is required to elucidate the additional functions that MCM10 plays in tumor growth and progression.

Therefore, our pan-cancer analysis based on data from public databases suggests that MCM10 may act as a novel prognostic biomarker as well as a potential therapeutic target. Figure S1 illustrates the process of this study.

## Materials and methods

### MCM10 expression profiles in human cancers

Data on MCM10 gene expression in tumor and healthy tissues were gathered using the Cancer Genome Atlas (TCGA, http://cancergenome.nih.gov) and genotype-tissue expression (GTEx, http://commonfund.nih.gov/GTEx/) databases. The complete names of each form of cancer are included in Table 1, along with their acronyms. MCM10 expression in human malignancies and associated normal tissue were compared using the TIMER database (https://cistrome.shinyapps.io/timer/)^14^. GEPIA’s “Stage plots” module was used to examine the relationship between MCM10 and cancer stage (gepia2.cancer-pku.cn/#index)^15^. Nevertheless, we applied TISIDB to analyze the link between MCM10 expression and immune and molecular subtypes of various kinds of cancer^16^.

**Table 1:** TCGA cancer abbreviations and the corresponding cancer type.

Abbreviations	Cancer type
ACC	Adrenocortical carcinoma
BLCA	Bladder urothelial carcinoma
BRCA	Breast invasive carcinoma
CESC	Cervical squamous cell carcinoma and endocervical adenocarcinoma
CHOL	Cholangiocarcinoma
COAD	Colon adenocarcinoma
DLBC	Lymphoid neoplasm diffuse large B-cell lymphoma
ESCA	Esophageal carcinoma
GBM	Glioblastoma multiforme
HNSC	Head and neck squamous cell carcinoma
KICH	Kidney chromophobe
KIRC	Kidney renal clear cell carcinoma
KIRP	Kidney renal papillary cell carcinoma
LAML	Acute myeloid leukemia
LGG	Brain lower grade glioma
LIHC	Liver hepatocellular carcinoma
LUAD	Lung adenocarcinoma
LUSC	Lung squamous cell carcinoma
MESO	Mesothelioma
OV	Ovarian serous cystadenocarcinoma
PAAD	Pancreatic adenocarcinoma
PCPG	Pheochromocytoma and paraganglioma
PRAD	Prostate adenocarcinoma
READ	Rectum adenocarcinoma
SARC	Sarcoma
SKCM	Skin cutaneous melanoma
STAD	Stomach adenocarcinoma
TGCT	Testicular germ cell tumors
THCA	Thyroid carcinoma
THYM	Thymoma
UCEC	Uterine corpus endometrial carcinoma
UCS	Uterine carcinosarcoma
UVM	Uveal melanoma

The levels of MCM10 protein expression in tumor tissues were assessed using a tool called Human Protein Atlas (HPA) (https://www.proteinatlas.org/)^17^. The tissue atlas and pathology atlas panels had images of MCM10 protein immunohistochemistry that we were able to locate.

### Diagnostic and prognostic analysis

The data of the mRNA expression of MCM10 in cancer and normal tissues in TCGA were utilized to determine the diagnostic value of MCM10 in pan-cancer using the receiver operating characteristic (ROC) curve. The ROC curves were calculated using the “pROC” (v1.17.0.1) package, and the “ggplot2” (v3.3.3) tool was used to plot the results. AUC > 0.7 was thought to be a reliable diagnostic marker. The association between MCM10 expression and prognosis was investigated by entering “MCM10” into the gene expression prognostic analysis under the pan-cancer analysis module on the Sangerbox website (http://sangerbox.com/Tool), selecting samples from the TCGA database, and removing samples with “0” expression^18^.

### Mutation profiles

A database of cancer genomics datasets available for free is the cBioPortal for cancer genomics (http://www.cbioportal.org)^19^. The distribution of MCM10 in various cancers was determined after entering the name of the MCM10 gene into the “Quick Search Beta!” interface. Gene copy number, mutation type, and frequency were among these factors.

### Methylation and copy number variation (CNV) analysis

Using the GSCALite platform (http://bioinfo.life.hust.edu.cn/web/GSCALite/), different TCGA cancer types' MCM10 methylation patterns in tumors and healthy tissues were studied^20^. Using the same methodology, we then dug into the connection between MCM10 methylation and its expression and prognosis in other cancers. In addition, we also used this platform to analyze the expression of MCM family genes (MCM1-10) associated with CNV in pan-cancer.

### Immune infiltration

TIMER database is a comprehensive tool for the systematic examination of immune infiltrates in distinct cancer types. We filled in the gene “MCM10” under the Immunity module in the TIMER database, selected the tumor type as well as the immune cell type, and in turn analyzed and obtained a scatter plot of the correlation between MCM10 expression and immune cell infiltration.

### Tumor mutational burden (TMB), microsatellite instability (MSI), immune checkpoints (ICPs) and tumor dryness analysis

We calculated the TMB and MSI for each tumor using the TMB function of the R package, integrated the TMB, MSI and gene expression data of the samples, and furthermore log2(x + 0.001) transformed each expression value and calculated their pearson correlation in each tumor. Additionally, we have examined the relationship between MCM10 expression and immunological checkpoints, DNAss tumor dryness score based on R package.

### Protein–protein interaction (PPI) network and enrichment analysis

The GEPIA database was used to retrieve the top 100 genes differentially expressed with MCM10 in pan-cancer and we subsequently performed PPI network construction via STRING database^21^ and GO/KEGG enrichment analysis on these genes. In order to make the PPI network diagram more aesthetically pleasing, we further tweaked it using the cytoscape software.

### CancerSEA analysis

The functional status of MCM10 in several cancer types was evaluated by CancerSEA (http://biocc.hrbmu.edu.cn/CancerSEA/)^22^. It consists of 14 functional states of 41,900 single cancer cells in 25 different types of malignancies. The cutoff for the MCM10 and cancer functional status correlation was determined at correlation ≥ 0.4 and *p* value < 0.05. The T-SNE graphs demonstrated the expression patterns of MCM10 at single cells.

### Drug sensitivity prediction

We conducted a drug sensitivity analysis of MCM10 using the oncoPredict R package based on the GDSC database, and the “ggplot2” (v3.3.3) package was used for plotting.

### Statistical analysis

Student's t test was used to determine the statistical significance of group differences, and one-way ANOVA was used to compare groups. Statistics were significant when the P-value was less than 0.05.

## Results

### Expression levels of MCM10 in different types of human cancers

**Figure 1:**
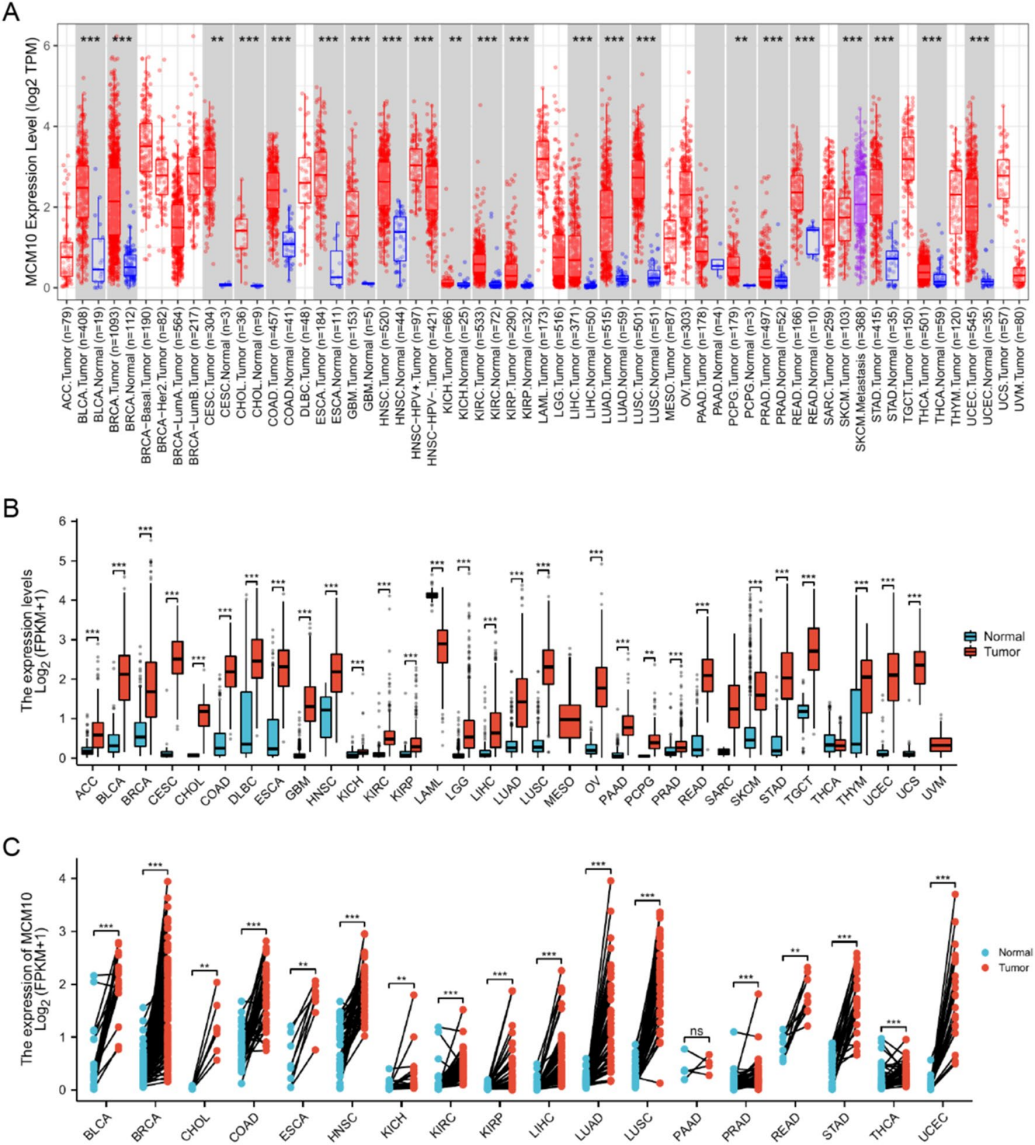
MCM10 mRNA expression levels in pan-cancer. (**A**) MCM10 expression levels in pan-cancer in the TIMER2.0 database. (**B**) TCGA and GTEx data suggest differential expression of MCM10 in a pan-cancer perspective. (**C**) TCGA data set paired MCM10 expression in cancerous tissues and adjacent normal tissues. *p < 0.05;**p < 0.01; ***p < 0.001.

**Figure 2:**
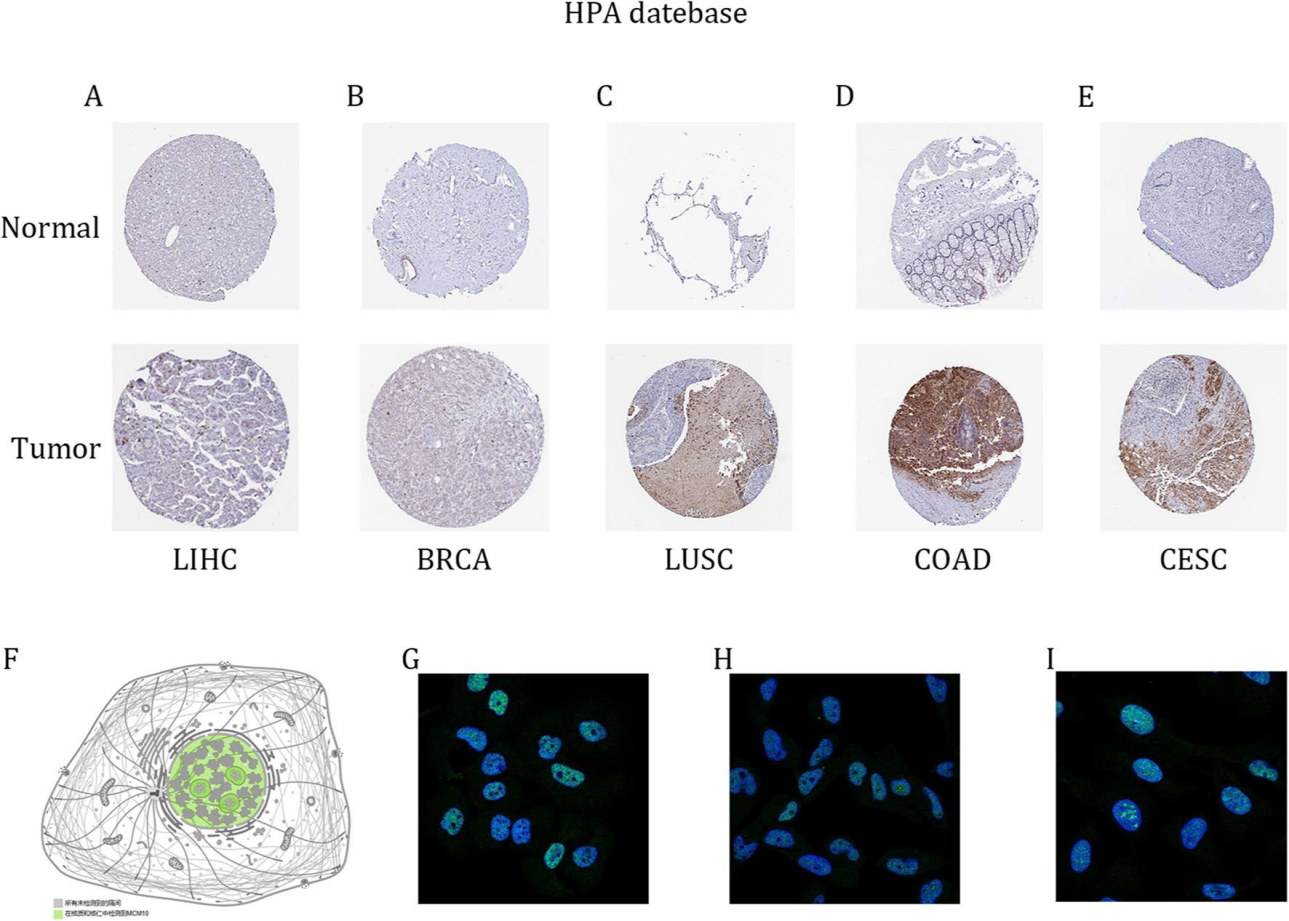
Protein expression levels and subcellular localization of MCM10 based on the HPA database. The immunohistochemical images of MCM10 protein expression in (**A**) LIHC; (**B**) BRCA; (**C**) LUSC; (**D**) COAD; (**E**) CESC. (**F**–I) Simulated plots and immunofluorescence plots of the subcellular localization of MCM10.

First of all, we examined MCM10 expression in the TCGA dataset using the TIMER 2.0 database to investigate the levels of MCM10 expression. In most malignancies, including BLCA, BRCA, CESC, CHOL, COAD, ESCA, GBM, HNSC, KICH, KIRC, KIRP, LIHC, LUAD, LUSC, PRAD, READ, STAD, THCA, and UCEC, the data revealed that MCM10 was considerably elevated (Fig. 1A). Compared to initial tumors, MCM10 expression was increased in metastatic cutaneous melanoma tumors. We incorporated information from the TCGA and GTEx databases to supplement the expression of MCM10 in pan-cancer samples because the TCGA database lacked normal samples (Fig. 1B). The outcomes revealed that there were also substantial variations in expression in ACC, DLBC, LAML, LGG, OV, PAAD, SKCM, TGCT, THYM, and UCS. Additionally, we explored MCM10 expression levels in matched samples (Fig. 1C). We further compared the expression levels of MCM10 in LIHC, BRCA, LUSC, COAD and CESC with those in the corresponding normal tissues through the HPA database (Fig. 2A–E). On the other hand, we queried the subcellular localization of MCM10 through the HPA database (Fig. 2F–I) and found that MCM10 was localized in the nucleoplasm and nucleolus, which agrees with previous literature^23^. According to the aforementioned findings, MCM10 expression is dysregulated in a number of tumor types, with most cancers expressing it substantially more than normal tissue does.

**Figure 3:**
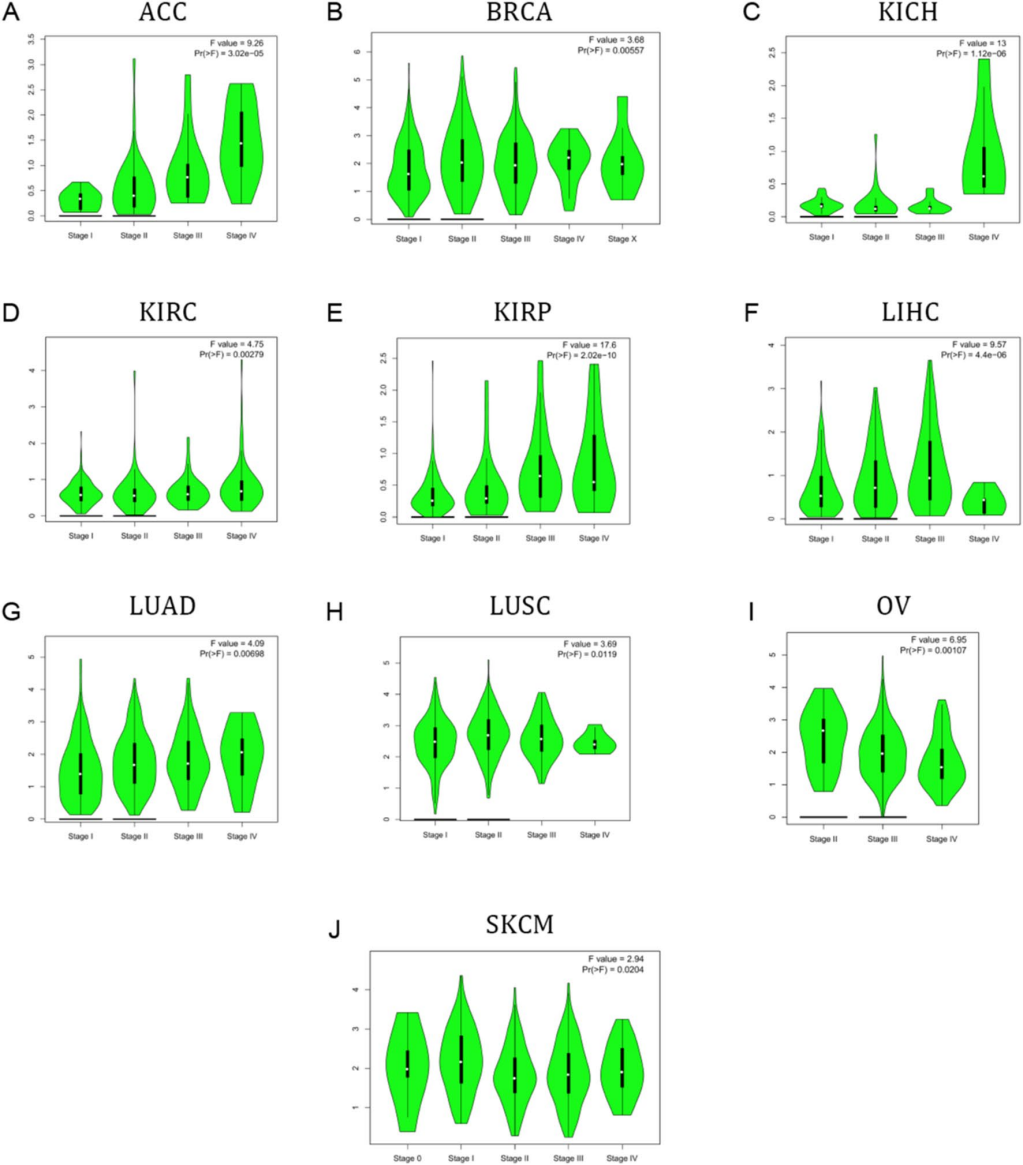
Major pathological stages of MCM10 expression based on the GEPIA2 database in (**A**) ACC; (**B**) BRCA; (**C**) KICH; (**D**) KIRC; (**E**) KIRP; (**F**) LIHC; (**G**) LUAD; (**H**) LUSC; (**I**) OV; (**J**) SKCM.

We also evaluated MCM10 expression in relation to pan-cancer tumor stage. The results emphasize relationship between MCM10 expression and the ACC, BRCA, KICH, KIRC, KIRP, LIHC, LUAD, LUSC, OV, and SKCM stages (Fig. 3). These findings imply that MCM10 expression levels are related to the clinical staging of these cancers and may play a role in determining the pathological staging of these malignancies.

Then, using the TISDB database, we examined the expression of MCM10 in distinct cancer immune and molecular subtype. Cancer immune (Figure S2A–G) and molecular subtype (Figure S2H–N) were shown to be substantially linked with MCM10 expression.

### The diagnostic and prognostic value of MCM10 in pan-cancer

The ROC curve was then used to examine the diagnostic utility of MCM10 in various malignancies. MCM10 may function as the ideal diagnostic marker in BLCA (AUC = 0.894), BRCA (AUC = 0.936), CESC (AUC = 1.000), CHOL (AUC = 1.000), CAOD (AUC = 0.955), ESCA (AUC = 0.960), GBM (AUC = 1.000), HNSC (AUC = 0.901), KICH (AUC = 0.762), KIRC (AUC = 0.944), KIRP (AUC = 0.931), LIHC (AUC = 0.948), LUAD (AUC = 0.970), LUSC (AUC = 0.996), PAAD (AUC = 0.736), PCPG (AUC = 0.989), PRAD (AUC = 0.706), READ (AUC = 0.941), STAD (AUC = 0.945), THCA (AUC = 0.753) and UCEC (AUC = 0.974) (Figure S3). MCM10 showed good diagnostic potential (AUC > 0.7) in many of the above cancer types, notably AUC = 1 in CESC, CHOL and GBM.

**Figure 4:**
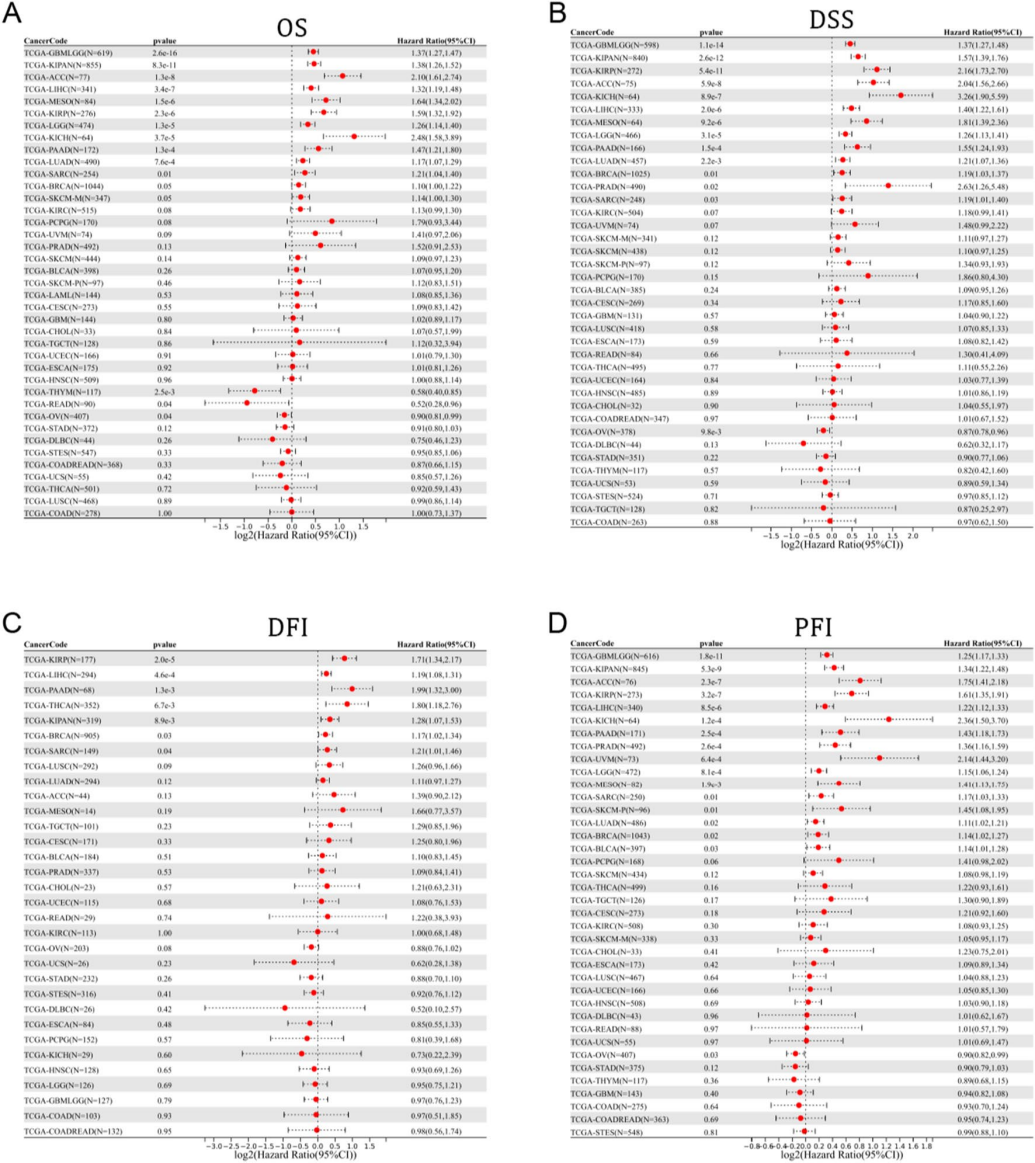
Prognostic value of MCM10 in pan-cancer. The results were shown with a forest map for (**A**) OS; (**B**) DSS; (**C**) DFI; (**D**) PFI.

After that, we investigated the correlation between pan-cancer patient OS and MCM10 expression. Through analysis of 32 cancer types, it was finally observed that in 12 tumor types (GBMLGG, LGG, LUAD, SARC, KIRP, KIPAN, LIHC, MESO, SKCM-M, PAAD, ACC and KICH) with poor prognosis of high expression, in three tumor types (THYM, READ and OV) with poor prognosis in low expression (Fig. 4A**)**.

We further explored the interrelation between MCM10 expression and Disease-specific survival (DSS) in patients with pancytopenia eventually observed in 13 tumor types (GBMLGG, LGG, LUAD, BRCA, SARC, KIRP, KIPAN, PRAD, LIHC, MESO, PAAD, ACC and KICH) with a poor prognosis for high expression and a poor prognosis for low expression in OV, as shown in Fig. 4B.

As for Disease-free interval (DFI), it was finally observed in seven tumor types (BRCA, SARC, KIRP, KIPAN, LIHC, THCA and PAAD) with poor prognosis in high expression (Fig. 4C).

For the progression-free interval (PFI), the final results were observed in 16 tumor types (GBMLGG, LGG, LUAD, BRCA, SARC, KIRP, KIPAN, PRAD, LIHC, MESO, PAAD, SKCM-P, UVM, BLCA, ACC and KICH) with a poor prognosis for high expression and a poor prognosis for low expression in OV (Fig. 4D). The corresponding survival curves are shown in Figures S4 and S5.

Our findings suggest that in the great majority of tumor types, increased MCM10 expression is linked to a poor prognosis. In contrast, a worse prognosis was linked to its low expression in OV.

### The methylation analysis of MCM10 in human cancers

DNA methylation is significantly linked to alterations in cancer gene expression, according to research^24^. Therefore, utilizing the GSCALite platform, the changes in DNA methylation between tumor and normal tissue in different malignancies were evaluated for MCM1-10. The analysis indicated that MCM10 methylation was highly up-regulated in LUAD and significantly down-regulated in KIRC, COAD, and PRAD (Figure S6A). The relationship between MCM10's DNA methylation and its mRNA expression in pan-cancer was then examined. The findings demonstrated that in the majority of tumors, MCM10 mRNA expression was primarily negatively correlated with its DNA methylation (Figure S6B). Additionally, we discovered a correlation between the OS of LGG and UVM and MCM10 methylation (Figure S6C). Notably, MCM10 mRNA expression and methylation were significantly correlated across multiple cancer types (Figure S7A), and further analysis of the correlation between MCM10 methylation and prognosis revealed that MCM10 methylation was associated with prognosis in a variety of cancers, especially in CHOL, and with OS, PFS, DFI, and DSS (Figure S7B). Taken together, MCM10 methylation is dysregulated in several cancer types. It may be used as a diagnostic tool for these types of cancers.

### The mutation landscape of MCM10 in human cancers

The frequency of MCM10 modifications (mutations and CNAs) in 32 TCGA cancer types was examined using the cBioPortal program to analyze the mutational landscape of MCM10 in human malignancies. The findings demonstrated that tumor type affected the frequency of MCM10 mutations. MCM10 was discovered to have a high modification frequency of close to 7% in BLCA, as illustrated in Figure S8A. Next, it was looked at how MCM10's putative CNA and its gene expression in cancers related to one another. In pan-cancer, the putative CNA of MCM10 was demonstrated in (Figure S8B,C). In conclusion, MCM10 mutations may be closely associated with tumorigenesis.

### MCM10 CNV in human cancers

The GSCALite platform’s copy number variation (CNV) module offered heterozygous and homozygous CNV profiles for 33 different cancer types. The pie chart demonstrated that different malignancies were more likely to have heterozygous CNVs in MCM1-10 (Figure S9A). Only BLCA, UCS and OV had homozygous amplification for MCM10; no homozygous deletion was discovered (Figure S9B).

MCM10 heterozygous amplification was common in most tumors (Figure S9C). Additionally, the relationship between CNV and gene expression was explored. The findings demonstrated that MCM10 expression was significantly and favorably linked with CNV in the majority of cancer types (Figure S9D). As a result, the expression of MCM10 may be directly related to CNV.

### Correlation of MCM10 expression with immune cell infiltration

**Figure 5:**
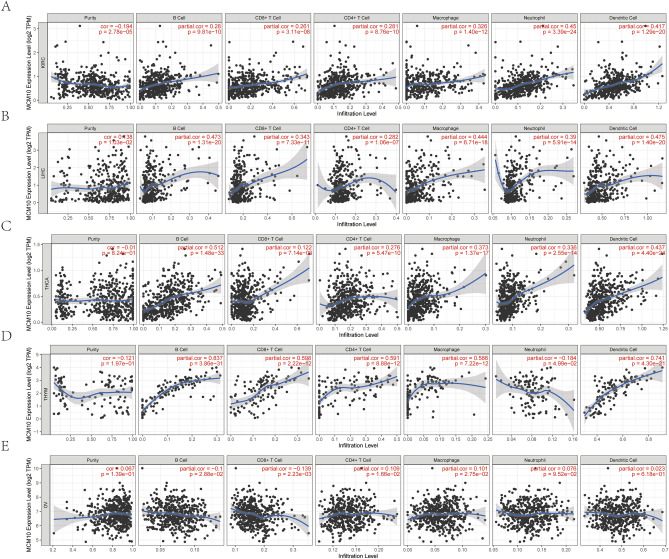
Correlation of MCM10 expression with immune infiltration in (**A**) KIRC; (**B**) LIHC; (**C**) THCA; (**D**) THYM; (**E**) OV.

The connection between MCM10 expression and particular immune cell infiltration in human cancer was also investigated. In KIRC, neutrophils and dendritic cells were substantially positively connected with MCM10 expression (Fig. 5A), while in LIHC, B cells, macrophages, and dendritic cells were favorably correlated (Fig. 5B). It had a stronger correlation with B cells and dendritic cells in THCA (Fig. 5C). However, its expression was substantially linked with dendritic cells, macrophages, CD8^+^ T cells, CD4^+^ T cells and B cells in THYM (Fig. 5D). In ovarian cancer, MCM10 expression, on the other hand, showed a weak correlation with immune cell infiltration, which may be one of the reasons why MCM10 expression in ovarian cancer seems to show opposite effects related to patient survival (Fig. 5E). In summary, MCM10 has a variety of impacts on immunity to various tumor types in the tumor microenvironment and may play a role in mediating the tumor immune response.

### MCM10 expression is related To TMB, MSI and tumor dryness in human cancers

Effective immune responses, which are encouraged in the setting of tumor growth, have been predicted by TMB and MSI. We also evaluated the strength of the association between TMB or MSI and MCM10 expression in pan-cancer. We found substantial positive associations with TMB in 14 tumors, including the following: ACC, BLCA, BRCA, COAD, LGG, LUAD, LUSC, OV, PAAD, PRAD, READ, SARC, STAD, THYM (Figure S10A). Then, we observed that MCM10 expression was significantly correlated with MSI in 9 tumors, including significant positive correlations in 7 tumors, such as: ACC, CESC, COAD, LIHC, LUSC, SARC, STAD. It was significantly negatively correlated in four tumors, e.g.: DLBC, THCA (Figure S10B). As for the correlation between MCM10 expression and tumor dryness, it was significantly correlated in 15 tumors, among which it was significantly positively correlated in 14 tumors, such as: GBM, LGG, CESC, LUAD, BRCA, ESCA, SARC, STAD, PRAD, HNSC, LUSC, LIHC, PAAD, TGCT and significantly negatively correlated in significant negative correlation in 1 tumor, e.g.: THYM (Figure S10C). These would suggest that MCM10 expression levels impact TMB and MSI in cancer, which in turn affects how well a patient responds to immune checkpoint inhibitor medication.

### Correlation of MCM10 expression with immune checkpoints in human cancers

Growing data suggests that immunological checkpoints (ICPs) play a major role in immunotherapy and tumor invasion^25^. The connection between MCM10 expression and ICPs expression in pan-cancer was investigated to ascertain the potential of MCM10 as a target for tumor immunotherapy. MCM10 expression was favorably connected with ICP expression in the majority of malignancies, particularly in KIRC, KIRP, LIHC, and UVM, while MCM10 expression and the majority of immunological checkpoints were also positively correlated (Figure S11A). The chordal plots further highlight the relationship between MCM10 expression in these four tumors and a number of significant immunological checkpoints (TLR4, CTLA4, VEGFA, CD276, CD274 BTN3A1 and BTN3A2) (Figure S11B–E). MCM10 may be a good target for tumor immunotherapy and may be significant in tumor immunotherapy, according to the significant association between MCM10 and immunological checkpoints. To further explore the response of MCM10 to cancer immunotherapy, we analyzed the immunotherapy cohort data IMvigor210 and showed that high MCM10 expression was more efficacious for PD-L1 treatment (Figure S11F).

### Enrichment analysis of MCM10-related biological functions

**Figure 6:**
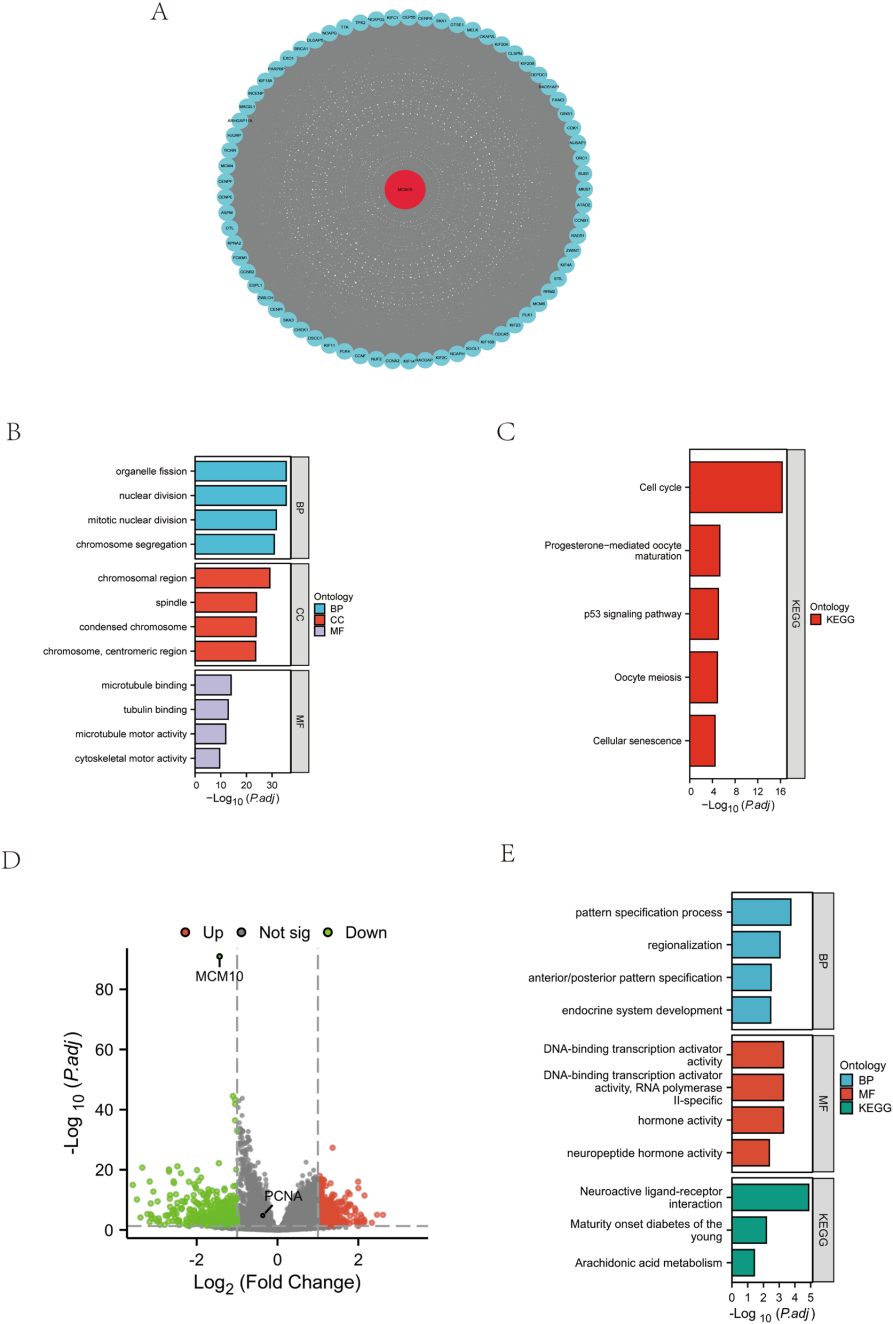
Functional annotation of MCM10 in pan-cancer. (**A**) Protein–protein interactions (PPI) network. (**B**) GO enrichment analysis. (**C**) KEGG enrichment analysis. (**D**) differentially expressed genes in ovarian cancer. (**E**) Enrichment analysis of differentially expressed genes in ovarian cancer.

We speculate that MCM10 is an oncogene in a number of cancer types and might thus be employed as a prognostic marker in light of our findings. The precise biochemical mechanism through which MCM10 promotes cancer is still a mystery. We created a list of the top 100 genes most closely related to MCM10 from GEPIA2. We imported these genes into the STRING database, constructed a PPI network, and adapted it using Cytoscape software (Fig. 6A). Then, we used GO and KEGG enrichment analysis to analyze the genes in each collection. The GO enrichment results showed that the biological functions of MCM10 were mainly related to organelle fission and nuclear division (Fig. 6B). The KEGG enrichment analysis suggested that MCM10 may affect tumor progression mainly by influencing cell cycle and P53 signaling pathways (Fig. 6C). Since MCM10 expression in ovarian cancer seems to show opposite effects related to patient survival, we analyzed and enriched genes with differential MCM10 expression in ovarian cancer (Fig. 6D). It was found to be mainly associated with the Neuroactive ligand-receptor interaction and Arachidonic acid metabolism pathways (Fig. 6E).

The impact of this gene on the activity of the tumor pathway was then investigated using GSCALite. The pie chart demonstrated that MCM10 activated DNA damage repair, cell cycle, and apoptosis while inhibiting the Hormone AR, RAS-MAPK, and RTK pathways (Figure S12A). Additionally, Figure S12B summarizes the interaction map of gene and pathway in which the mRNA expression of MCM1-10 may influence pathway activity.

### Single-cell analysis

**Figure 7:**
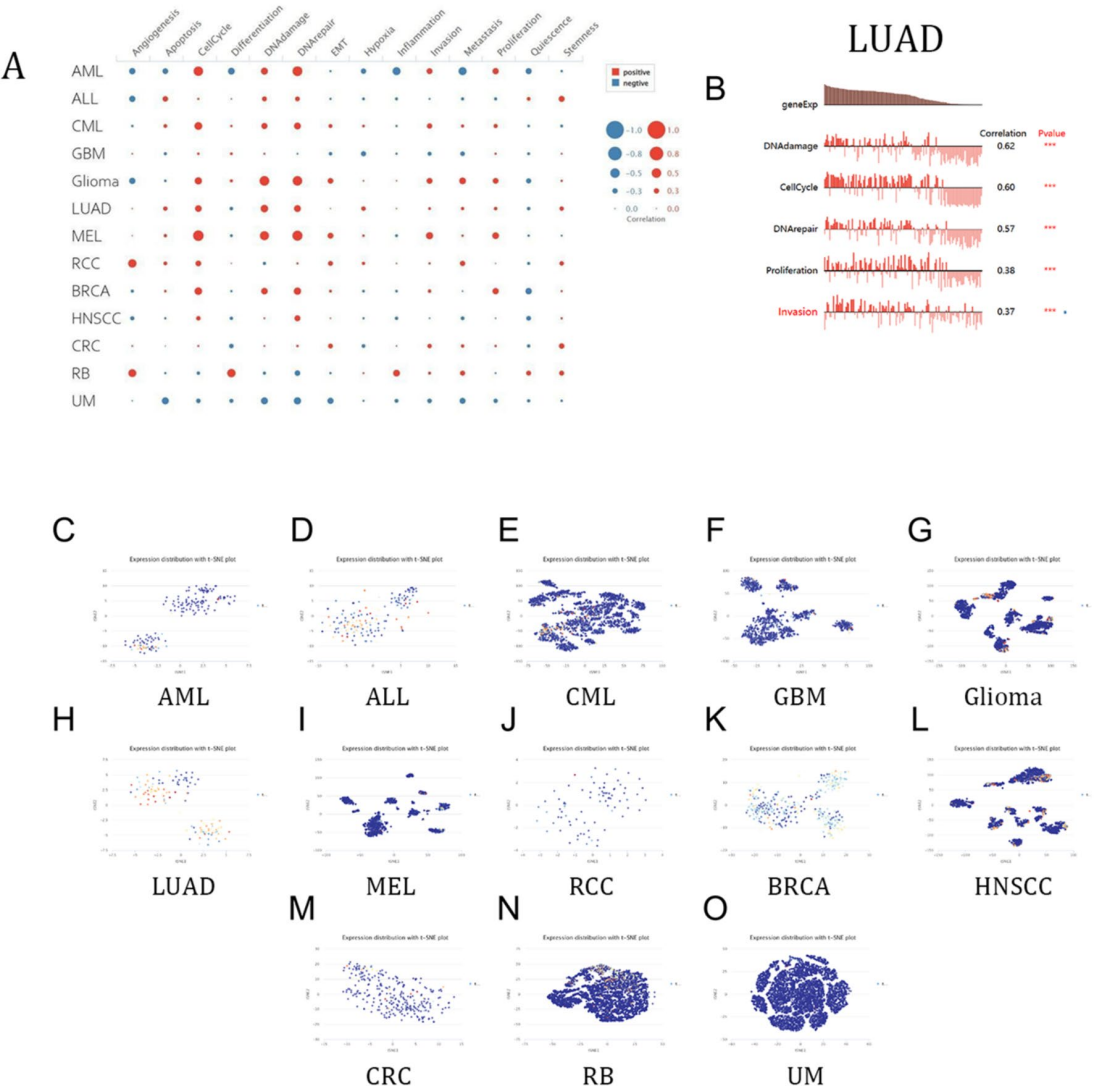
Single-cell sequence data analysis from the CancerSEA database. (**A**) Correlation of MCM10 expression with 14 kinds of functional status in 13 cancers. (**B**) The most predominantly enriched functional states in LUAD. (**C**–**O**) The T-SNE maps of MCM10 expression patterns in single cells.

The analysis of single cells' transcriptomes is a crucial technique for understanding various cancers, immune cells, endothelial cells, and stromal cells^26–28^. Analysis of the data from the CancerSEA database suggested us that MCM10 expression was significantly associated with the biological processes of cell cycle, DNA damage and repair, and EMT in the 13 cancers in the figure (Fig. 7A). In LUAD, it was most predominantly associated with DNA damage and cell cycle with correlation coefficients of 0.62 and 0.60, respectively (Fig. 7B). We also display the T-SNE maps of MCM10 expression patterns in single cells from the following tumor types: AML, ALL CML, GBM, glioma, AST, LUAD, MEL, RCC, BRCA, HNCC, CRC, RB, and UM (Fig. 7C–O). All of the aforementioned information suggests that MCM10 is crucial to the molecular mechanisms governing tumor incidence and progression.

### Monitoring for possible drug

**Figure 8:**
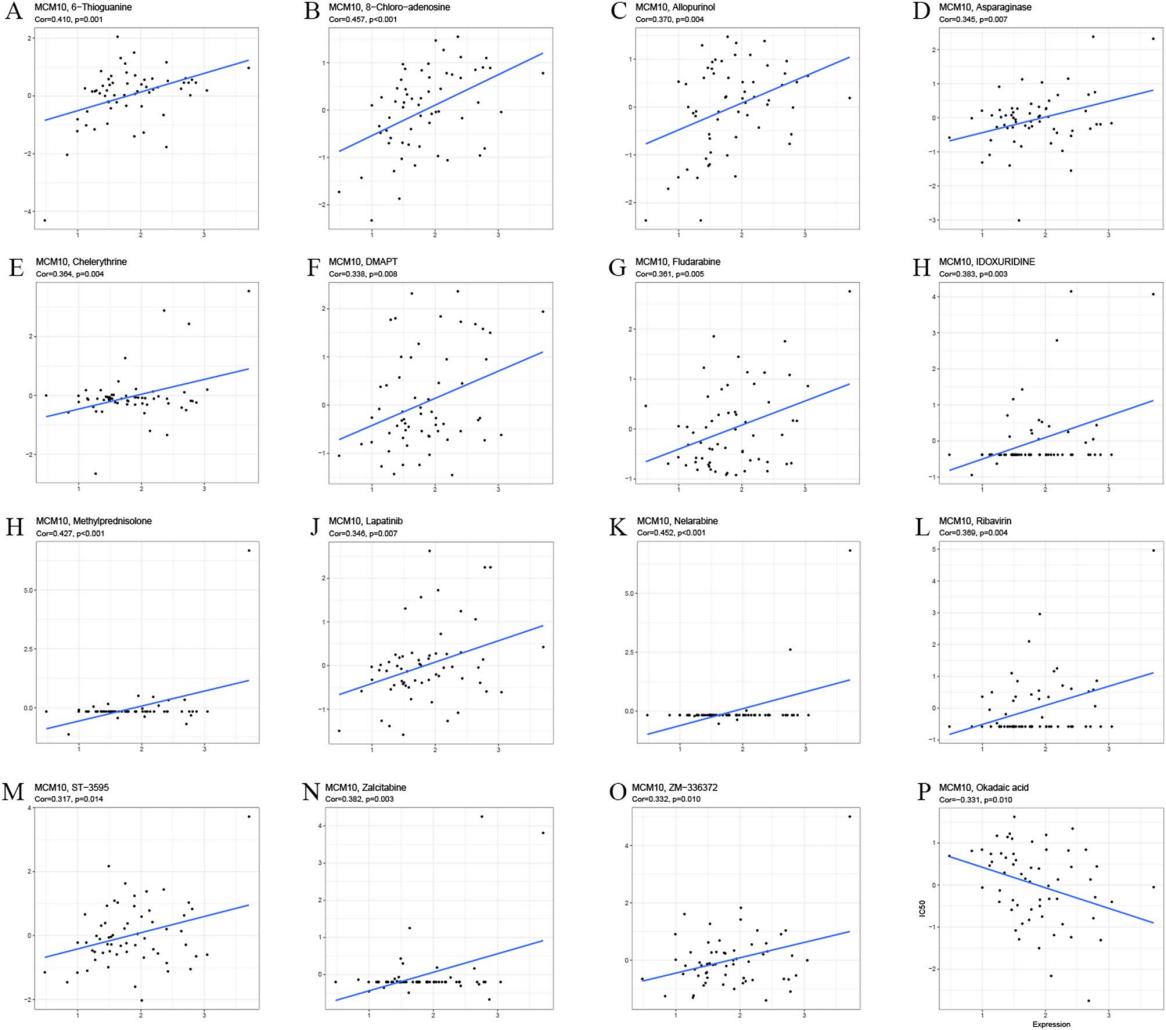
Correlation of MCM10 expression with small molecule/drug sensitivity.

To identify potential therapeutic medicines associated with MCM10 expression, we performed a Spearman correlation analysis of MCM10 expression and drug sensitivity (IC50). The outcomes demonstrated that MCM10 expression was inversely linked with the 50% inhibitory concentration (IC50) of most small molecule drugs such as 6-Thioguanine, 8-Chloro-adenosine, Allopurinol, Asparaginase, Chelerythrine, DMAPT, Fludarabine, IDOXURIDINE, Lapatinib, Methylprednisolone, Nelarabine, Ribavirin, ST-3595, Zalcitabine, ZM-336372. In contrast, only Okadaic acid was positively correlated with MCM10 expression (Fig. 8). Based on sensitivity analysis, high levels of MCM10 showed high resistance to several drugs, suggesting that they may serve as biomarkers for screening drugs.

## Discussion

In eukaryotes, the MCM complex regulates the process of DNA replication, and the equilibrium between nascent and parental MCM proteins stabilizes genome replication^29,30^. Abnormalities in this process can lead to tumorigenesis. MCM10 plays a crucial role in this process^31^.

A recent study suggests that excessive activation of MCM10 promotes genomic instability in early breast cancer^32^. Our study also found that MCM10 expression was significantly upregulated in most cancer types, which may contribute to tumorigenesis. Additionally, malignancies including breast cancer^33^, osteosarcoma^34^, and squamous lung carcinoma^35^ have poor prognoses when MCM10 is highly expressed. Further research demonstrated that MCM10 is strongly linked to immune cell infiltration, immunological subtypes, and molecular subtypes in several cancers. Our study also found that MCM10 has diagnostic value in most cancer types, particularly in CESC, CHOL, and GBM.

Immune checkpoint molecules normally downregulate co-stimulatory molecule activation signals to preserve self-tolerance and avoid autoimmunity. The state of T lymphocytes' functionality and activation can be suppressed by tumor cells through this method, which results in failure of T cells and tumor immune evasion^36,37^. As a result, we looked further into the relationship between MCM10 and immunological checkpoints. We discovered that MCM10 had substantial relationships with several immune checkpoints, which suggests that MCM10 could act as a novel immune checkpoint for tumor immunity.

Effective identification of possible tumor antigens can aid in the development of cancer immunotherapy, which is essential for the treatment of cancer. Most frequently, these copy number mutations or methylation alterations are linked to these tumor antigens^38,39^. The mutational landscape of MCM10 in pan-cancer is shown in our work, along with a landscape of connection with copy number variation and methylation. These findings serve as a benchmark for MCM10's effectiveness as an immunological indicator as well as a TME indicator.

Finally, we confirmed the function of MCM10 as a possible target for tumor therapy by examining the link between MCM10 expression and drug sensitivity. This indicated prospective therapeutic agents.

Notably, in some cancer types, such as OV, READ and THYM, patients with high MCM10 expression instead have a better prognosis, showing an opposite trend to other cancer types. Moreover, in ovarian cancer, MCM10 expression was also weakly correlated with immune cell infiltration, which may be related to immune escape from the tumor. This aroused great interest in us, and thus we are also conducting related experiments to deeply explore the potential role of MCM10 in ovarian cancer and whether it may serve as a potential therapeutic target.

This study is still constrained even if the pan-cancer analysis offers extensive information. The majority of the findings are based on data analysis, and additional experimental validation is required to offer convincing proof. As a result, more experimental research is still required to ascertain if MCM10 can be used as a target for cancer therapy.

## Conclusions

Overall, using a variety of techniques, we determined the importance of MCM10 in pan-cancer. As a potential target for tumor treatment and a promising prognostic and diagnostic biomarker for immunomodulation, MCM10 exhibits promise.

### Supplementary Information

Supplementary Figures.

## Data Availability

Transcriptome data and related clinical data of 33 cancers were downloaded from TCGA through the UCSC Xena platform (https://xenabrowser.net/datapages/). Other data can be found in the HPA database (http://www.proteinatlas.org/), TISIDB database (http://cis.hku.hk/TISIDB/), CancerSEA (http://biocc.hrbmu.edu.cn/CancerSEA/) and GSCALite platform (http://bioinfo.life.hust.edu.cn/web/GSCALite/). All data is publicly available.

## References

[1] Freddie B, Jacques F, Isabelle S (2018). Global cancer statistics 2018: GLOBOCAN estimates of incidence and mortality worldwide for 36 cancers in 185 countries. CA Cancer J. Clin..

[2] Bi GCZ, Yang X, Liang J, Hu Z, Bian Y, Sui Q (2020). Identification and validation of tumor environment phenotypes in lung adenocarcinoma by integrative genome-scale analysis. Cancer Immunol. Immunother..

[3] Santucci C, Carioli G, Bertuccio P, Malvezzi M, Vecchia CL (2020). Progress in cancer mortality, incidence, and survival: A global overview. Eur. J. Cancer Prev..

[4] Feng C, Wendl MC, Wyczalkowski MA, Bailey MH, Lie Y, Li D (2021). Moving pan-cancer studies from basic research toward the clinic. Nat. cancer.

[5] Samra JS, Jamieson NB, Gill AJ, Biankin A, Chang D (2020). Pan-cancer analysis of whole genomes. Nature.

[6] Huang R, Zhou PK (2021). DNA damage repair: Historical perspectives, mechanistic pathways and clinical translation for targeted cancer therapy. Signal Transduct. Target Ther..

[7] Merchant AM, Kawasaki Y, Chen Y, Lei M, Tye BK (1997). A lesion in the DNA replication initiation factor Mcm10 induces pausing of elongation forks through chromosomal replication origins in *Saccharomyces cerevisiae*. Mol. Cell. Biol..

[8] Solomon NA, Wright MB, Chang S, Buckley AM, Gaber RF (1992). Genetic and molecular analysis of DNA43 and DNA52: Two new cell-cycle genes in *Saccharomyces cerevisiae*. Yeast.

[9] Yeeles JTP, Janska A, Deegan TD (2015). Regulated eukaryotic DNA replication origin firing with purified proteins. Nature.

[10] Ravikiran M, Henrique N, Shun Y, Muhammad J, Yuchen B, Hiu-Fung Y (2018). DNA replication licensing protein MCM10 promotes tumor progression and is a novel prognostic biomarker and potential therapeutic target in breast cancer. Cancers.

[11] Wan W, Shen Y, Li Q (2020). MCM10 acts as a potential prognostic biomarker and promotes cell proliferation in hepatocellular carcinoma: Integrated bioinformatics analysis and experimental validation. Cancer Manag. Res..

[12] Wu Z, Wang Y, Li J, Wang H, Tuo X, Zheng J (2022). MCM10 is a prognostic biomarker and correlated with immune checkpoints in ovarian cancer. Front. Genet..

[13] Murayama T, Takeuchi Y, Yamawaki K, Natsume T, Li M, Marcela RN (2021). MCM10 compensates for Myc-induced DNA replication stress in breast cancer stem-like cells. Cancer Sci..

[14] Li T, Fan J, Wang B, Traugh N, Chen Q, Liu JS (2017). TIMER: A web server for comprehensive analysis of tumor-infiltrating immune cells. Cancer Res..

[15] Tang Z, Kang B, Li C, Chen T, Zhang Z (2019). GEPIA2: An enhanced web server for large-scale expression profiling and interactive analysis. Nucleic Acids Res..

[16] Ru B, Wong CN, Tong Y, Zhong JY, Zhong SSW, Wu WC (2019). TISIDB: An integrated repository portal for tumor-immune system interactions. Bioinformatics.

[17] Uhlén M, Björling E, Agaton C, Szigyarto CA-K, Amini B, Andersen E (2005). A human protein atlas for normal and cancer tissues based on antibody proteomics. Mol. Cell. Proteomics.

[18] Shen W, Song Z, Zhong X, Huang M, Shen D, Gao P (2022). Sangerbox: A comprehensive, interaction-friendly clinical bioinformatics analysis platform. iMeta.

[19] Gao J, Aksoy BA, Dogrusoz U, Dresdner G, Gross B, Sumer SO (2013). Integrative analysis of complex cancer genomics and clinical profiles using the cBioPortal. Sci. Signal.

[20] Liu C-J, Hu F-F, Xia M-X, Han L, Zhang Q, Guo A-Y (2018). GSCALite: A web server for gene set cancer analysis. Bioinformatics.

[21] von Mering C, Huynen M, Jaeggi D, Schmidt S, Bork P, Snel B (2003). STRING: A database of predicted functional associations between proteins. Nucleic Acids Res..

[22] Yuan H, Yan M, Zhang G, Liu W, Deng C, Liao G (2019). CancerSEA: A cancer single-cell state atlas. Nucleic Acids Res..

[23] Izumi M, Yatagai F, Hanaoka F (2004). Localization of human Mcm10 is spatially and temporally regulated during the S phase. J. Biol. Chem..

[24] Moore LD, Le T, Fan G (2013). DNA methylation and its basic function. Neuropsychopharmacology.

[25] Topalian SL, Taube JM, Anders RA, Pardoll DM (2016). Mechanism-driven biomarkers to guide immune checkpoint blockade in cancer therapy. Nat. Rev. Cancer.

[26] Lim B, Lin Y, Navin N (2020). Advancing cancer research and medicine with single-cell genomics. Cancer Cell.

[27] Lei Y, Tang R, Xu J, Wang W, Zhang B, Liu J (2021). Applications of single-cell sequencing in cancer research: Progress and perspectives. J. Hematol. Oncol..

[28] Li P-H, Kong X-Y, He Y-Z, Liu Y, Peng X, Li Z-H (2022). Recent developments in application of single-cell RNA sequencing in the tumour immune microenvironment and cancer therapy. Mil. Med. Res..

[29] Sedlackova H, Rask M-B, Gupta R, Choudhary C, Somyajit K, Lukas J (2020). Equilibrium between nascent and parental MCM proteins protects replicating genomes. Nature.

[30] Bailis JM, Forsburg SL (2004). MCM proteins: DNA damage, mutagenesis and repair. Curr. Opin. Genet. Dev..

[31] Baxley RM, Bielinsky A-K (2017). Mcm10: A dynamic scaffold at eukaryotic replication forks. Genes (Basel).

[32] Mughal MJ, Chan KI, Mahadevappa R, Wong SW, Wai KC, Kwok HF (2022). Over-activation of minichromosome maintenance protein 10 promotes genomic instability in early stages of breast cancer. Int. J. Biol. Sci..

[33] Tang J, Kong D, Cui Q, Wang K, Zhang D, Gong Y (2018). Prognostic genes of breast cancer identified by gene co-expression network analysis. Front. Oncol..

[34] Zhou J, Wang M, Zhou Z, Wang W, Duan J, Wu G (2021). Expression and prognostic value of MCM family genes in osteosarcoma. Front. Mol. Biosci..

[35] Wang M, Xie S, Yuan W, Xie T, Jamal M, Huang J (2019). Minichromosome maintenance protein 10 as a marker for proliferation and prognosis in lung cancer. Int. J. Oncol..

[36] Diesendruck Y, Benhar I (2017). Novel immune check point inhibiting antibodies in cancer therapy-opportunities and challenges. Drug Resist. Updat..

[37] Kumar P, Saini S, Prabhakar BS (2020). Cancer immunotherapy with check point inhibitor can cause autoimmune adverse events due to loss of Treg homeostasis. Semin. Cancer Biol..

[38] Pardoll DM (2012). The blockade of immune checkpoints in cancer immunotherapy. Nat. Rev. Cancer.

[39] Schumacher TN, Schreiber RD (2015). Neoantigens in cancer immunotherapy. Science.

