# Supplementary material for: MCM10, a potential diagnostic, immunological, and prognostic biomarker in pan-cancer
Source: Sci Rep. 2023 Oct 17;13:17701. doi: 10.1038/s41598-023-44946-2 (PMC10582070; doi:10.1038/s41598-023-44946-2)
Supplement: Supplementary file 1 — Supplementary Figures. [file 41598_2023_44946_MOESM1_ESM.docx]

**Supplementary Figure**

**
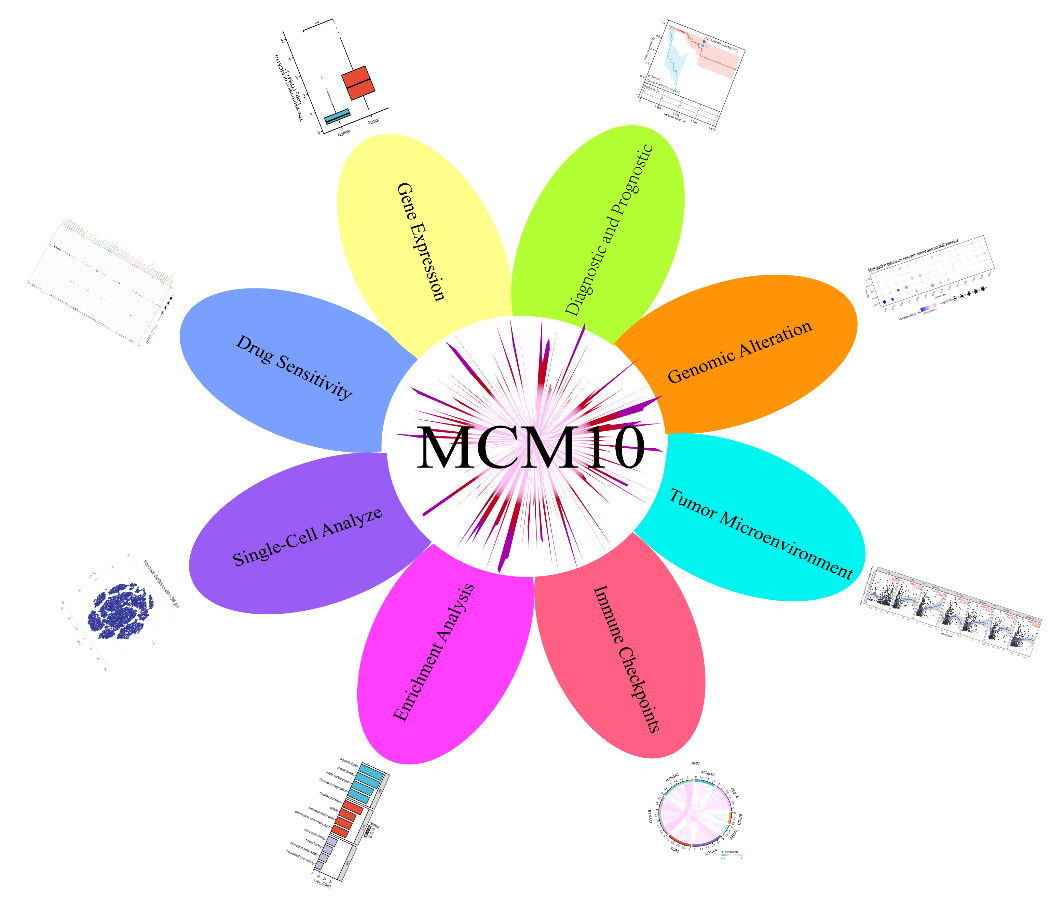
**

**Figure S1**

Flowchart for the study's process.

**
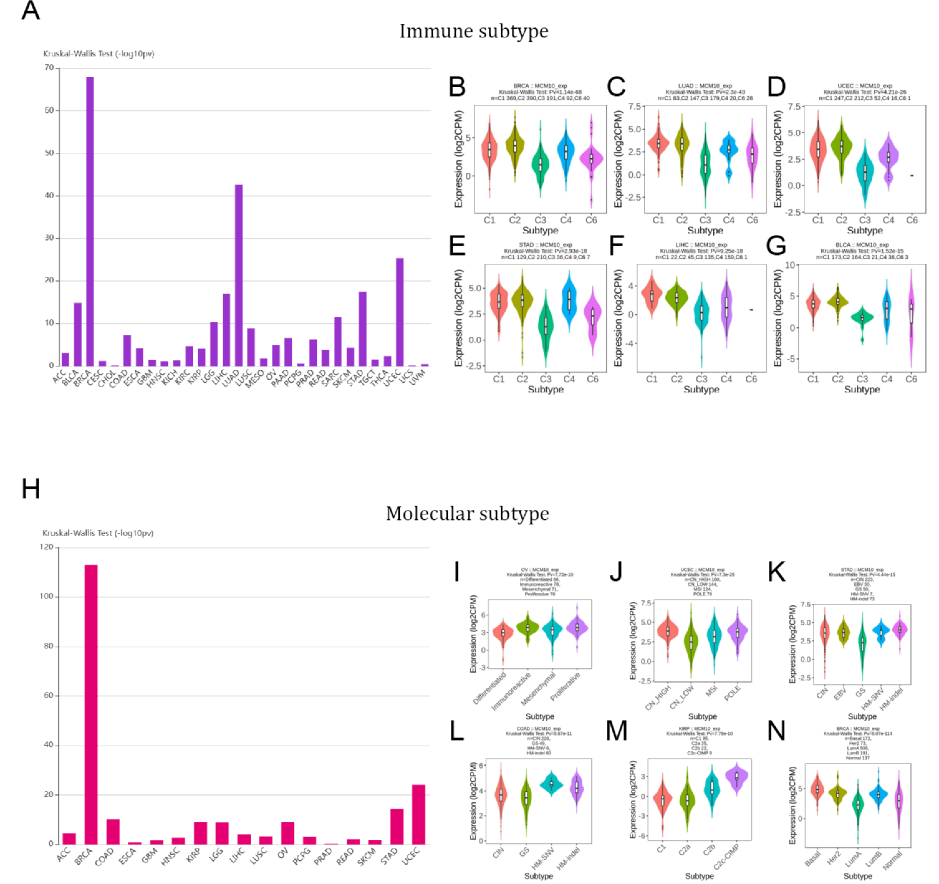
**

**Figure S2**

The expression of MCM10 in distinct cancer immune and molecular subtype. (A-G) Immune subtype; (H-N) molecular subtype. Six categories of immune subtypes were identified: C1 (wound healing), C2 (IFN-gamma dominant), C3 (inflammatory), C4 (lymphocyte deficient), C5 (immunologically silent), and C6 (TGF-b dominant).

**
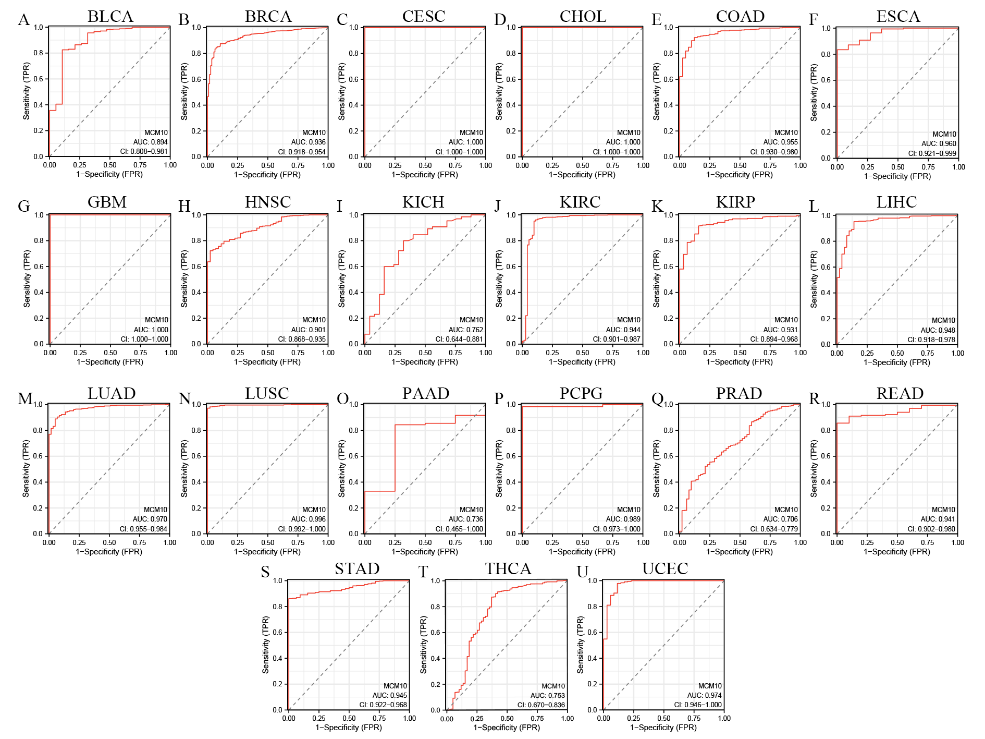
**

**Figure S3**

The diagnostic value of MCM10 to distinguish tumor tissue from normal tissue. (A) BLCA (AUC=0.894); (B) BRCA (AUC=0.936); (C) CESC (AUC=1.000); (D) CHOL (AUC=1.000); (E) CAOD (AUC=0.955); (F) ESCA (AUC=0.960); (G) GBM (AUC=1.000); (H) HNSC (AUC=0.901); (I) KICH (AUC=0.762); (J) KIRC (AUC=0.944); (K) KIRP (AUC=0.931); (L) LIHC (AUC=0.948); (M) LUAD (AUC=0.970). (N) LUSC; (O) PAAD; (P) PCPG; (Q) PRAD; (R) READ; (S) STAD; (T) THCA; (U) UCEC.


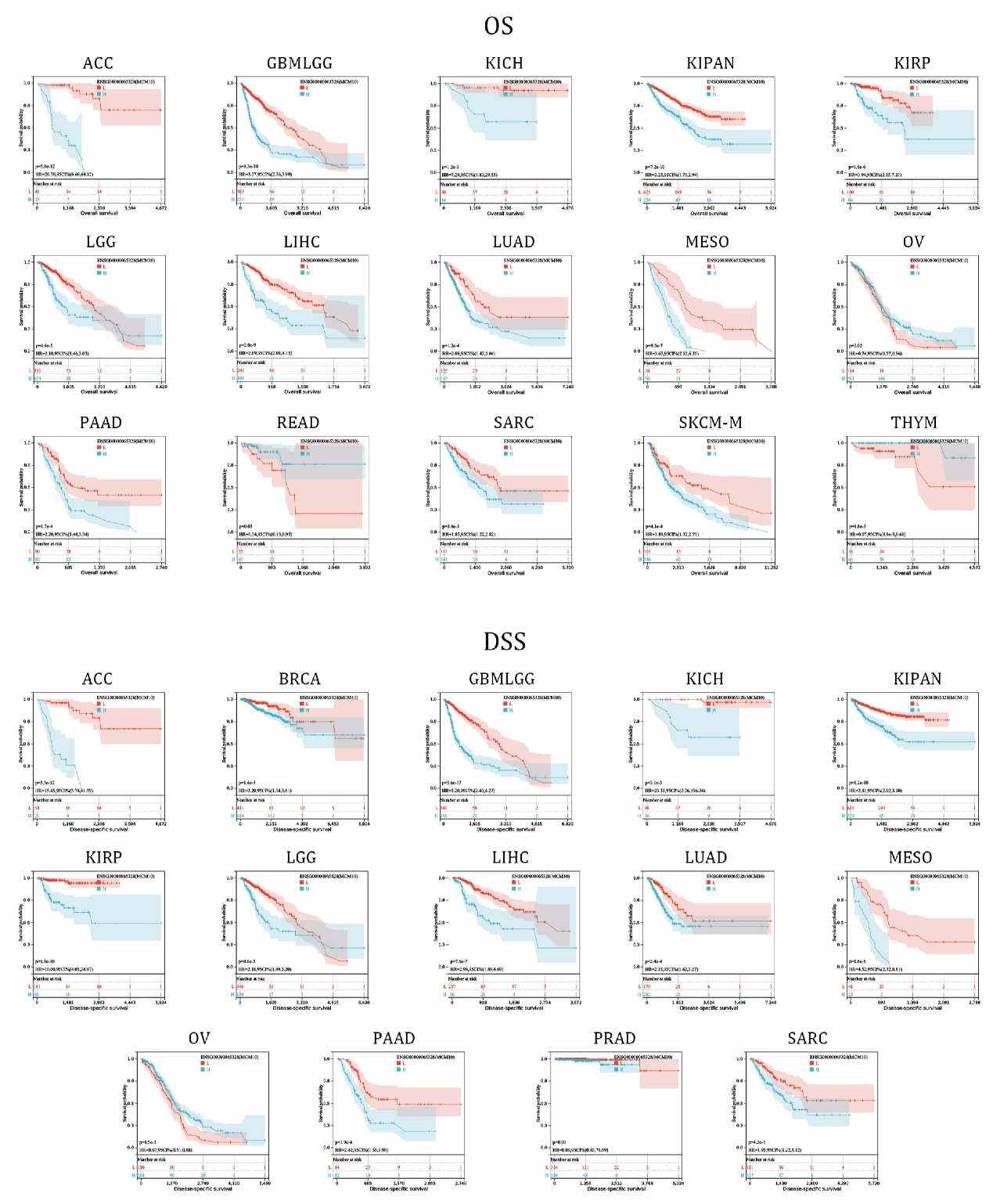


**Figure S4**

Survival curves of MCM10 expression and patient prognosis, including OS and DSS.


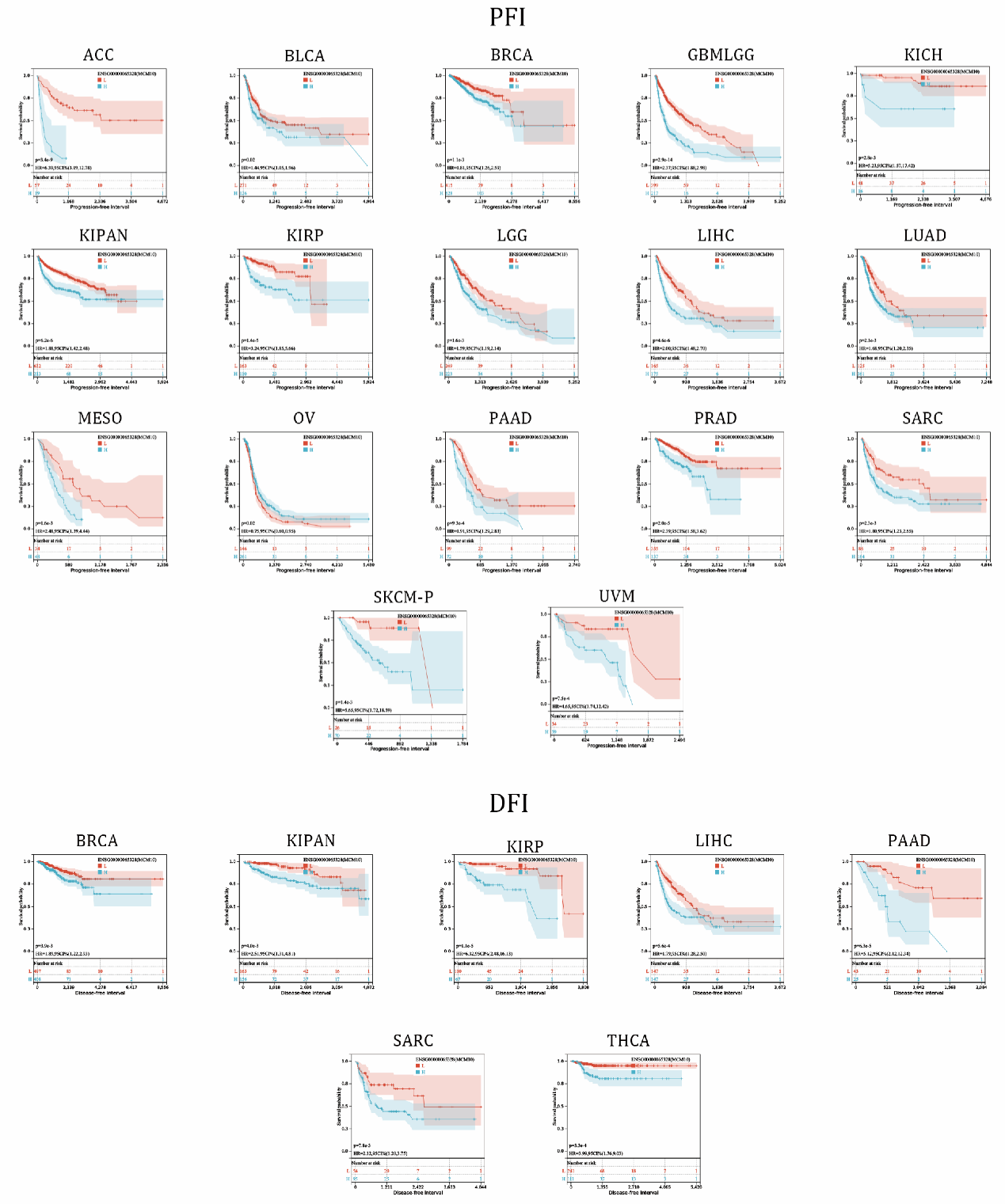


**Figure S5**

Survival curves of MCM10 expression and patient prognosis, including PFI and DFI.

**
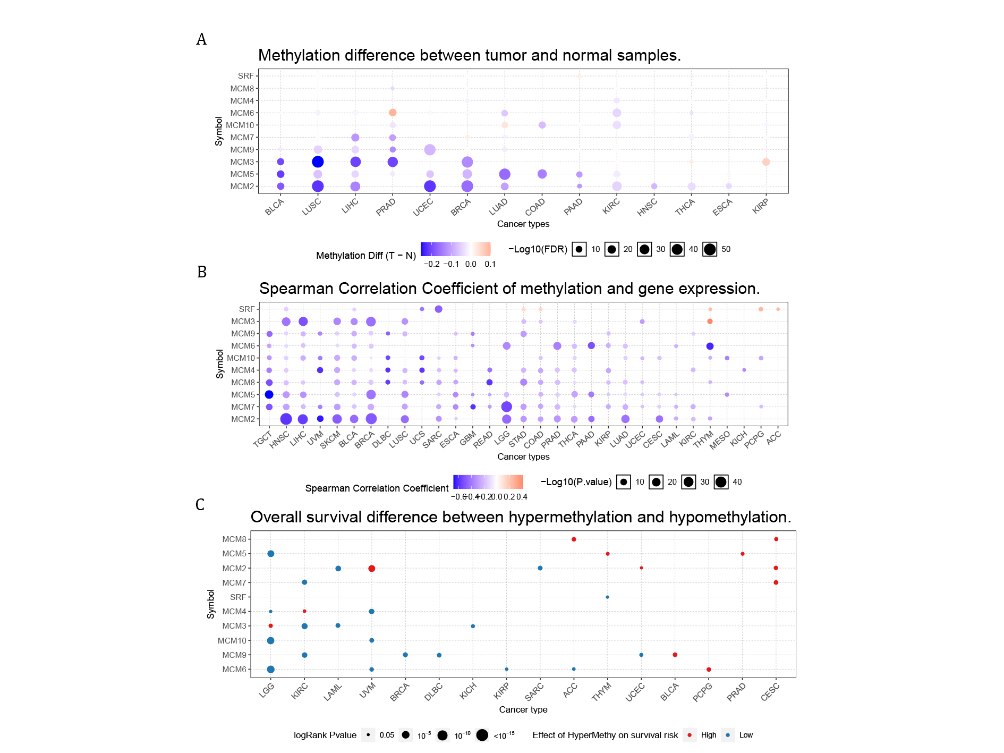
**

**Figure S6**

Methylation analysis and mutational mapping of MCM10 in human cancers. (A) DNA methylation differences of MCM10 between tumor and normal tissues in various cancers using the GSCALite platform. (B) Correlation between DNA methylation of MCM10 and mRNA expression in 32 cancer types. (C) Correlation between the OS and MCM10 methylation.


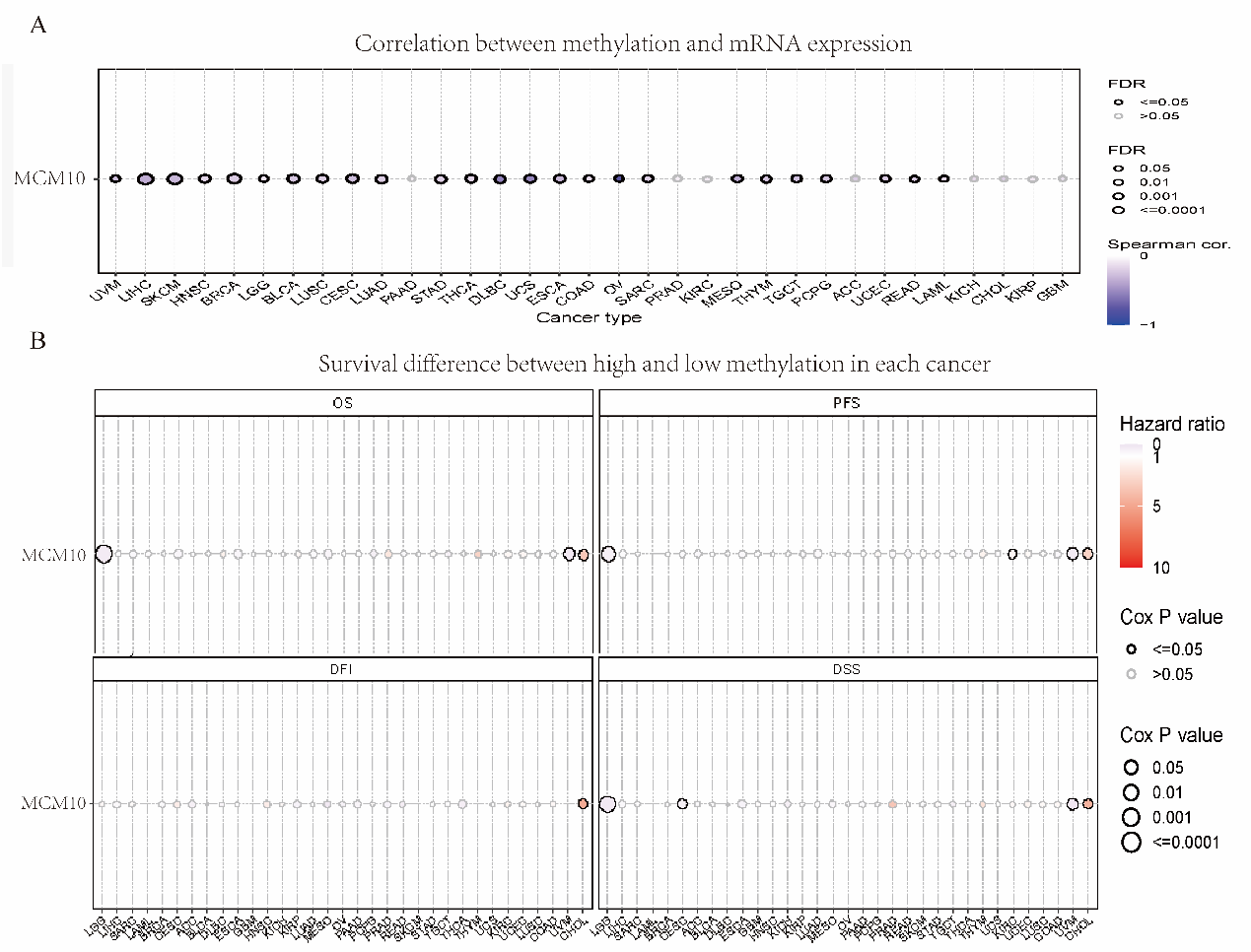


**Figure S7**

Methylation analysis of MCM10. (A) Correlation between mRNA expression and methylation of MCM10 in pan-cancer. (B) Correlation of MCM10 methylation with OS, PFS, DFI and DSS.

**
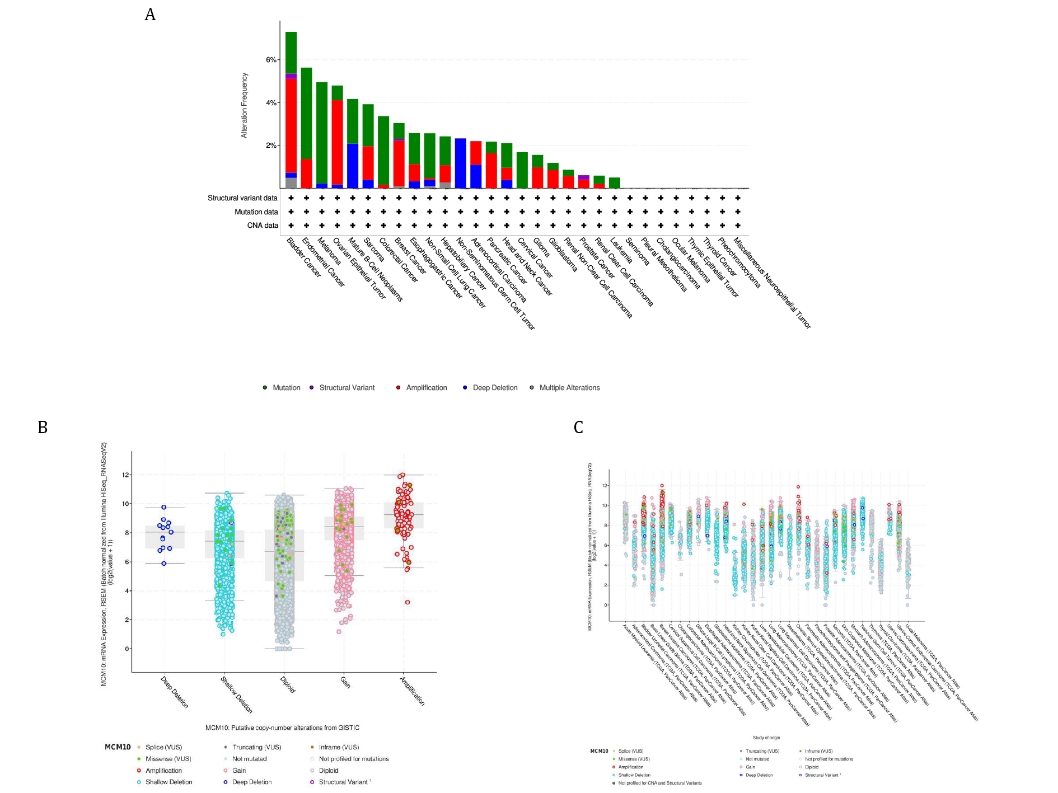
**

**Figure S8**

(A) Frequency of MCM10 alterations (mutations and CNA) in 32 TCGA cancer types. (B, C) Correlation between putative copy number alterations of MCM10 and its expression in tumors.

**
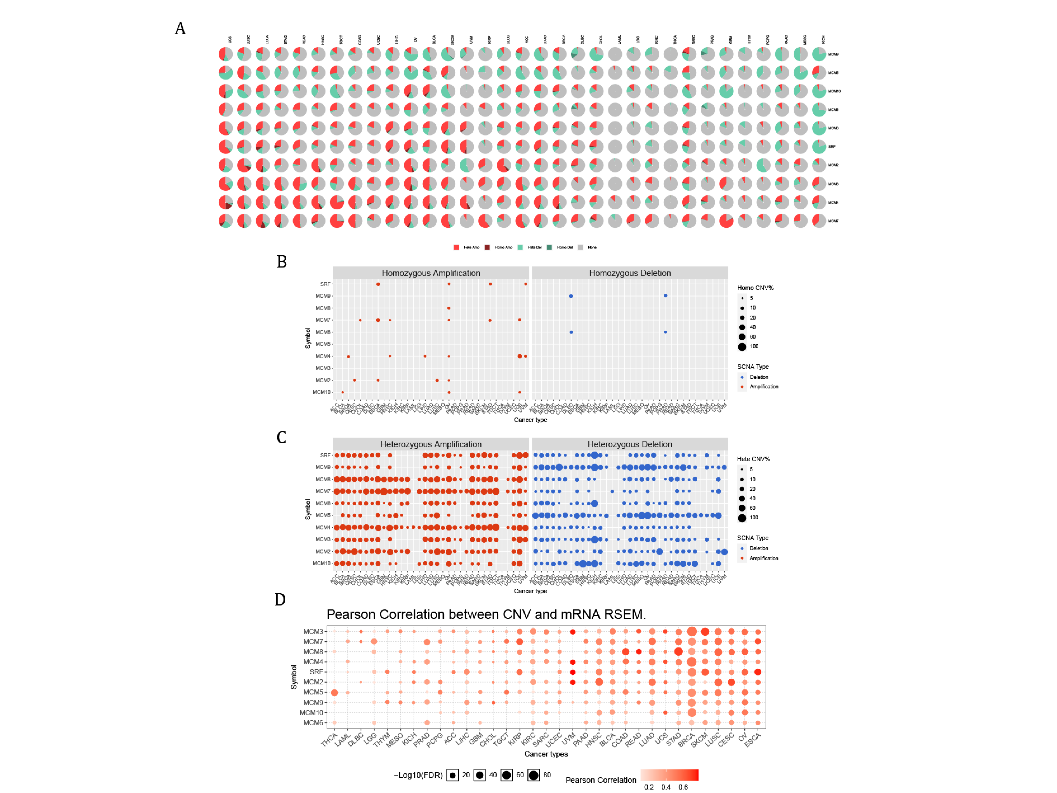
**

**Figure S9**

MCM10 CNVs in human cancers. (A) CNV profiles of 33 cancer types using the GSCALite platform for MCM1-10. Homo Amp stands for homozygous amplification, whereas Hete Amp and Hete Del stand for heterozygous amplification and deletion, respectively. (B) Pancytopenia with homozygous amplified or absent CNVs of MCM1-10. The size of the bubbles indicates the fraction of CNVs, with blue bubbles denoting deletions and red bubbles denoting amplifications. (C) Pancytopenia with heterozygous amplification or deletion of the CNV for MCM1-10. (D)Correlation between CNV and the mRNA expression of MCM1-10. Red and blue bubbles denote positive and negative correlations, respectively; the deeper the hue, the stronger the association.

**
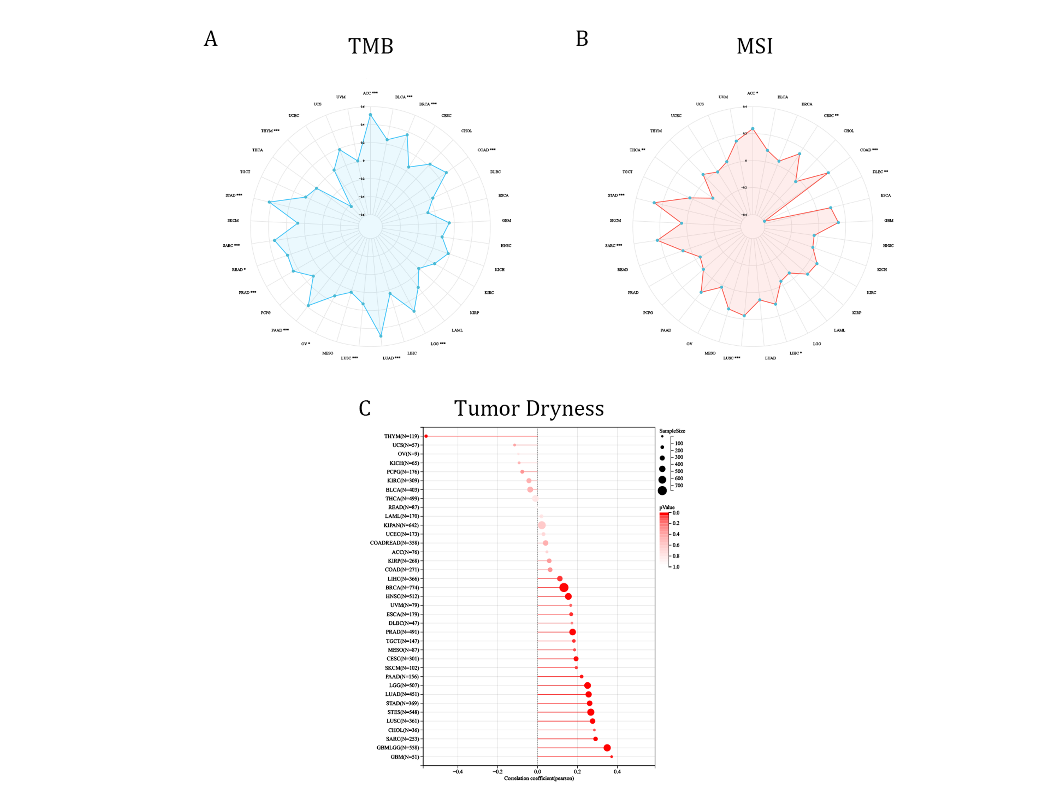
**

**Figure S10**

Association of MCM10 expression with TMB, MSI and tumor dryness in pan-cancer. (A) TMB; (B) MSI; (C) tumor dryness.

**
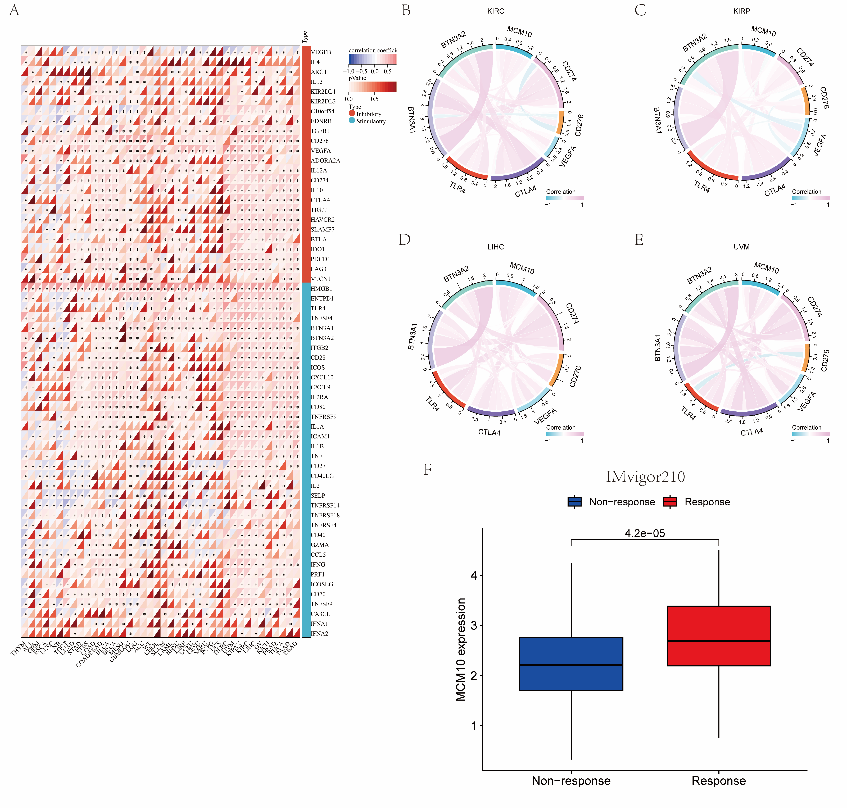
**

**Figure S11**

Correlations between MCM10 expression and ICPs. (A) Correlation of MCM10 expression with ICP gene levels in pan-cancer. The chordal plots further highlight the relationship between MCM10 expression and a number of significant immunological checkpoints (TLR4, CTLA4, VEGFA, CD276, CD274 BTN3A1 and BTN3A2) in (B) KIRC; (C) KIRP; (D) LIHC; (E) UVM. (F) MCM10 expression in response to PD-L1 treatment.

**
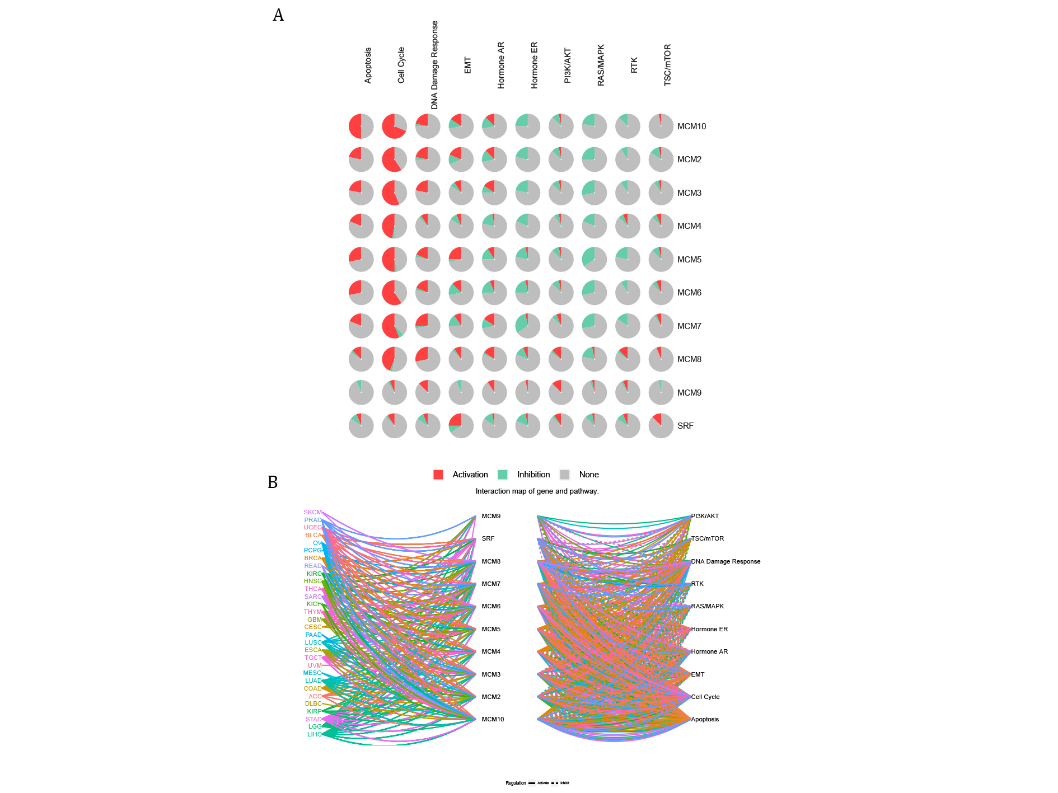
**

**Figure S12**

The impact of this gene on the activity of the tumor pathway. (A) Pie chart; (B) interaction map.
